# Paratyphoid Fever A: Infection and Prevention

**DOI:** 10.3389/fmicb.2022.945235

**Published:** 2022-07-08

**Authors:** Lei Xie, Lan Ming, Manlin Ding, Luxin Deng, Miao Liu, Yanguang Cong

**Affiliations:** ^1^Precision Medicine Center, The Affiliated Traditional Chinese Medicine Hospital of Southwest Medical University, Luzhou, China; ^2^Department of Clinical Laboratory, The Affiliated Traditional Chinese Medicine Hospital of Southwest Medical University, Luzhou, China; ^3^Department of Clinical Laboratory, The Affiliated Hospital of Southwest Medical University, Luzhou, China

**Keywords:** enteric fever, paratyphoid fever A, vaccine, bivalent vaccine, *Salmonella enterica* serovar Paratyphi A

## Abstract

Enteric fever is caused by *Salmonella enterica* serovar Typhi, *Salmonella enterica* serovar Paratyphi A, B, and C. While *S*. Typhi remains the primary causative agent of enteric fever, *S*. Paratyphi A is responsible for an increasing portion of enteric fever incidence. However, the current available vaccines for enteric fever are all developed from *S*. Typhi, and lack adequate cross immune protection against paratyphoid fever A. Therefore, paratyphoid A vaccines are urgently needed. The present paper reviews the latest progresses in pathogenesis, global burden, infection features of paratyphoid fever A, as well as the status of vaccine development, highlighting the necessity for the development of vaccines against paratyphoid fever A.

## Introduction

The *Salmonella* genus is composed of two species (*Salmonella enterica* and *Salmonella bongori*), six subspecies and more than 2,500 serovars. Most of medically related serovars belong to *S. enterica subspecies. enterica*, and cause multiple diseases from benign gastroenteritis to fatal systemic infection. Serovars Typhi and Paratyphi cause typhoid fever and paratyphoid fever, which are collectively known as enteric fever (Bhandari et al., 2022). Typhoidal *Salmonella* strains are restricted to humans except *S*. Paratyphi C. Non-typhoidal *Salmonella* (NTS), which has broad host range, usually leads to self-limiting diarrhea. However, when NTS strains invade the tissues, they result in febrile invasive diseases, such as bacteremia, meningitis, or focal infections, with high case fatality. In this instance, the strains were named invasive non-typhoidal *Salmonella* (iNTS) (Marchello et al., 2022).

*Salmonella* infections cause a huge global burden of morbidity and mortality. According to the data from the Institute for Health Metrics and Evaluation, 13.0 million cases of enteric fever occurred in 2019, resulting in 133 000 deaths (IHME, 2022a). The number of iNTS infection is 594 000 cases in 2019 with mortality of 13.3% (IHME, 2022b). Enteric fever is prevalent in low- and middle-income countries with poor sanitary conditions and lack of clean drinking water (Bhandari et al., 2022). Kim et al. (2019) collected and analyzed the global outbreak data of enteric fever from 1990 to 2018. There were altogether 303 identified outbreaks of typhoid and paratyphoid fever. Most of them occurred in Asia (51%), Africa (15%), and Oceania (14%) (Kim et al., 2019). In high-income countries, enteric fever is primarily associated with overseas travels, especially to high prevalence areas (Date et al., 2016; Muresu et al., 2020). The iNTS infection is predominant in sub-Saharan Africa (Balasubramanian et al., 2019), however, they also occurred in other regions of the world, for example, Asia (Le Thi Phuong et al., 2017; Southeast Asia Infectious Disease Clinical Research, 2017).

Effective control of enteric fever depends on comprehensive measures including health education, improving sanitation and supply of clean drinking water. However, implementation of these measures is hardly achieved in a short term in the low-income areas due to economic limitation. In the latest WHO position paper on typhoid vaccines, WHO reemphasized the importance of vaccination, and recommended the use of a new generation of typhoid conjugate vaccines to control enteric fever (World Health and Organization, 2019).

All typhoid *Salmonella* serovars belong to one subspecies, and are highly similar. These serovars share a set of antigens. For example, vi polysaccharide presents in both *S*. Typhi and *S*. Paratyphi C (Hu et al., 2017). O-12 antigen is the common O antigen component of *S*. Typhi, *S*. Paratyphi A and B (Pakkanen et al., 2014). Furthermore, a number of protein antigens are shared by varied typhoid *Salmonella* serovars (Jesudason et al., 1991). These common antigens constitute the basis of cross immunoreactions between typhoid *Salmonella* serovars. However, the cross immunoreactions do not ensure sufficient immunoprotection to prevent the infection caused by heterologous serovars. For example, the live attenuated vaccine Ty21a conferred considerable efficacy in preventing infection caused by *S*. Paratyphi B in large clinical trials, while no significant protection against paratyphoid A was observed (Black et al., 1990; Simanjuntak et al., 1991; Levine et al., 1999).

*S*. Typhi remains the principal agent of enteric fever. However, the infection cases caused by *S*. Paratyphi A increased steadily in recent decades, and became a non-negligible causative serotype of enteric fever. There is no currently licensed vaccine specifically for the control of paratyphoid fever A so far. All the current vaccines of enteric fever, including Ty21a live attenuated vaccine, Vi polysaccharide vaccine and Vi conjugate vaccine, were developed from *S*. Typhi, and conferred inadequate cross protection against paratyphoid fever A. Therefore, paratyphoid A vaccines are urgently needed. Currently, multiple paratyphoid A vaccine candidates, including attenuated vaccine candidates, subunit vaccine candidates and vesicle vaccine candidates, are under development. In the present paper, we reviewed the latest research progresses of these paratyphoid A vaccine candidates, highlighting the necessity and open questions of paratyphoid A vaccines.

## The Global Burden of Paratyphoid Fever

The global incidence of paratyphoid fever (mostly paratyphoid fever A) was approximately 3.8 million cases in 2019, which accounted for 29.2% of the total incidence of enteric fever (typhoid and paratyphoid) (IHME, 2022c). An increasing trend of paratyphoid fever A has been reported over the past two decades (Sahastrabuddhe et al., 2013), specifically in Asia areas, including Nepal (Zellweger et al., 2017), Cambodia (Kuijpers et al., 2017), and China (Arndt et al., 2014). In some areas, *S*. Paratyphi A became the predominated serotype responsible for enteric fever (Jin, 2008; Yaxian et al., 2015; Kuijpers et al., 2017).

Another challenge for the control of enteric fever is the emergence and rapid spread of anti-microbial resistance. The resistant rates of isolates of *S*. Paratyphi A as well as *S*. Typhi to fluoroquinolone increased dramatically during the past decades (Yaxian et al., 2015; Browne et al., 2020; Rahman et al., 2021; Manoharan et al., 2022). Furthermore, the high incidence rate of enteric fever in epidemic areas commonly leads to substantial overtreatment of suspected cases with unnecessary antimicrobials to prevent possible serious consequences. That may result in increased anti-microbial resistance not only in typhoid *Salmonella* but also other pathogenic bacteria of febrile diseases, which are difficult to distinguish from enteric fever (Andrews et al., 2019). Fluoroquinolone-resistant *Salmonellae* have been ranked as high priority pathogens for the research and development of new antibiotics (Tacconelli et al., 2018).

## Infection and Protective Immunity

Human is the only natural host of enteric fever pathogens except *S*. Paratyphi C. Their genomes are notable for accumulation of a large number of pseudogenes, which reflect their host-restriction natures. There are 173 pseudogenes (4.1% of annotated coding sequences) in the Paratyphi A genome, and is slightly fewer than the number of the Typhi pseudogenes (approximately 210, ∼ 5% of annotated coding sequences). However, only 30 pseudogenes are shared in both genomes indicating that *S*. Paratyphi A and *S*. Typhi evolved independently (McClelland et al., 2004). Although the two serovar are closely related, they differ in production of certain virulence factors, for example, Vi polysaccharide capsule. The Vi polysaccharide serves as an important virulent factor as well as a protective antigen in *S*. Typhi, while is absent in *S*. Paratyphi A (Hu et al., 2017).

A clinical observation on *S*. Paratyphi A infection using paratyphoid human challenge model showed that pathogenesis of *S*. Paratyphi A is highly similar to that of *S*. Typhi (Dobinson et al., 2017). Humans are typically infected with *Salmonella* through ingesting contaminated foods or drinking water. *Salmonella* cells survive in the passage through the stomach to the intestine due to their acid resistance nature. In the distal ileum, pathogens invade intestinal epithelial cells, especially the specialized microfold cells (M cells), which mostly overlie the Peyer’s patches. After passing through the M cells, the bacteria enter the underlying structure of lymphoid tissue. From the initial infection site in the Peyer’s patches, the bacteria access the mesenteric lymph nodes, and finally enter the blood through the output lymphatic vessels. After disseminating to systemic tissues, *Salmonella* cells replicate in the phagocytes of spleen, liver and bone marrow, resulting in serious lesions (Manesh et al., 2021).

Similar to typhoid fever, paratyphoid fever is characterized by febrile illness, and in severe cases, gastrointestinal bleeding, altered mental status, intestinal perforation, and death. *S*. Paratyphi A is formerly assumed to cause a milder disease than does *S*. Typhi (Bhan et al., 2005), which is further supported by the recent research data using human challenge models (Waddington et al., 2014; Dobinson et al., 2017). The Paratyphi A challenge led to a milder symptom profile with fewer severe symptoms and lower cumulative symptom scores. However, a clinical observation in Nepal showed that the infections caused by the two serovar were clinically indistinguishable and had equal severity (Maskey et al., 2006).

The infecting dose required to induce paratyphoid disease is almost as same as the dose for *S*. Typhi to cause typhoid fever. In the studies using the human challenge model, an attack rate of 60% was achieved at an oral challenge dose of 1–5 × 10^3^ CFU of *S*. Paratyphi A following sodium bicarbonate pretreatment, which is comparable to the attack rate of 55% challenged with a dose of 1 × 10^3^ CFU of *S*. Typhi (Waddington et al., 2014; Dobinson et al., 2017). The findings showed high similarity between typhoid fever and paratyphoid fever A.

Host immunity against *Salmonella* infection includes innate immunity and acquired immunity. CD4 T cells play a major role in the protective immunity of primary and secondary *Salmonella* infection. In addition, natural immune cells, CD8 T cells and B cells also play important roles in the clearance of pathogens (Pham and McSorley, 2015). The important role of humoral immunity in the protective immunity against the infection caused by typhoid *Salmonella* has been a controversial topic for a long time. Now a common consensus is that antibodies are essential in the protective immunity against systemic *Salmonella* infection, and can generate the highest protective outcome by cooperating with cell-mediated immunity (Mastroeni and Rossi, 2020). Antigen specificity, isotype profile, FcγR receptor usage, and complement activation play important roles in the regulation of antibody-mediated protection to *Salmonella* (Dahora et al., 2019; Mastroeni and Rossi, 2020).

## The Necessity for the Development of Paratyphoid A Vaccines

As early as 1896, Richard Pfeiffer and Almroth Wright published their studies of heat-inactivated whole-cell vaccine, respectively (Williamson et al., 2021). The vaccine had been widely used during World War I, and determined to be efficacious (Shanks, 2014). However, the inactivated whole-cell vaccines usually caused severe local and systemic adverse events, and had been removed from the licensed vaccine list.

There are three currently licensed vaccines for enteric fever in the world, including oral live vaccine Ty21a, Vi subunit vaccine, and Vi conjugate vaccine. All these vaccines are developed from *S*. Typhi. Vaccination is effective and feasible in the control of enteric fever. For example, the incidence rate of enteric fever has decreased dramatically in recent decades in China, from 10 to 50 per 100,000 before 1990 to less than 1 per 100,000 nowadays (Dong et al., 2020), due to the large-scale administration of the Vi polysaccharide vaccine as well as improvement in sanitation and clean water supply. The effectiveness of typhoid vaccines was also confirmed by Vietnam’s achievements in the control of typhoid fever (Basnyat and Karkey, 2019). Therefore, the programmatic use of typhoid vaccines is recommended by the World Health Organization (WHO) for the prevention of enteric fever (World Health and Organization, 2019).

Due to absence of Vi in *S*. Paratyphi A, administration of Vi subunit vaccine and Vi conjugate vaccine do not confer immunoprotection against the infection caused by *S*. Paratyphi A. On the contrary, immunization of Vi-derived vaccines may lead to serovar conversion from *S*. Typhi to *S*. Paratyphi A in epidemic areas. For example, a sharp serovar conversion from *S*. Typhi to *S*. Paratyphi A was observed in Guangxi, a high endemic region of China, in 1998, following 3 years of use of the Vi subunit vaccine (Dong et al., 2010). All outbreaks prior to 1998 were caused by *S*. Typhi. However, more than 80% of enteric fever outbreaks were due to *S*. Paratyphi since 1998.

Data on efficacy of the live vaccine Ty21a against the infection caused by *S*. Paratyphi A are conflicting. Previous observations revealed that immunization with Ty21a induced circulating plasmablasts and multifunctional CD8 T-cell responses that cross reacted with *S*. Paratyphi A in humans, though the magnitude of the responses to *S*. Paratyphi A was lower than those to *S*. Typhi and *S*. Paratyphi B (Wahid et al., 2012, 2015). A small-scale observation in Israel showed that the immunization with Ty21a protected overseas travelers from the infection caused by *S*. Paratyphi A (Meltzer et al., 2005). However, large field studies in highly endemic areas showed that Ty21a had not adequate cross protection against infection caused by *S*. Paratyphi A (Black et al., 1990; Simanjuntak et al., 1991; Levine et al., 1999). Furthermore, a recent challenge-re-challenge study using a controlled human infection model provided convincing evidence for the low level of cross-immunoprotection between *S*. Typhi and *S*. Paratyphi A. In the study, heterologous re-challenge with *S*. Typhi or *S*. Paratyphi A was not associated with a reduced attack rate following challenge (Gibani et al., 2020).

So far there is no currently licensed vaccine available for the control of paratyphoid A fever. Given the increasing threaten of *S*. Paratyphi A and the inadequate cross-immunity between *S*. Typhi and *S*. Paratyphi A, development of paratyphoid A vaccines is urgently needed.

## Paratyphoid A Vaccines Under Development

### Live Attenuated Vaccine Candidates

Several target genes are frequently used for genetically engineered mutagenesis in construction of attenuated *Salmonella* strains, including *htrA*, *ssaV*, *phoP*/*phoQ*, *clpPX*, genes encoding aromatic acid biosynthesis pathway, etc. Some of their homologs in *S*. Paratyphi A have been evaluated in animal experiment or human volunteers.

#### *phoP*/*phoQ*

PhoP and PhoQ constitute a two-component regulatory system, which controls the expression of multiple virulence genes and is critical in *Salmonella* pathogenesis (Miller et al., 1989). Roland et al. (2010) constructed four *phoPQ*-deleted strains from different parent strains of *S*. Paratyphi A. The mutants were more sensitive to deoxycholate and polymyxin B than their parent strains. Of them, the mutant MGN10028 was demonstrated to be well-tolerated at all doses administered and immunogenic following a single oral inoculation in an oral rabbit model. Immunization with MGN10028 induced immune responses that protected animals against the clinical manifestations following the challenge of *S*. Paratyphi A (Roland et al., 2010).

#### *aroC* and *yncD*

Aromatic compounds are deficient in human body. Thus, deletion of genes involved in aromatic biosynthesis usually leads to dramatic attenuation. The *yncD* gene encodes a TonB-dependent transporter, and is also required for bacterial virulence (Xiong et al., 2012). Xiong et al. (2015) constructed an attenuated *S*. Paratyphi A strain, SPADD01, by deleting *aroC* and *yncD* (Xiong et al., 2015). The 50% lethal dose of the resultant mutant was approximately 40,000-fold higher than that of the parent strain. Moreover, SPADD01 showed high immunogenicity in mice model. Single intranasal vaccination of SPADD01 elicited high levels of humoral and mucosal immune responses, and conferred protection against lethal challenge of the wild-type strain (Xiong et al., 2015).

#### *htrA* and *yncD*

The encoding product of *htrA* is a stress response protease degrading misfolded or damaged proteins in the periplasmic space, and is required for bacterial survival under various stresses, for example, high temperature and oxidative shock (Strauch et al., 1989; Pallen and Wren, 1997). Deficiency of the HtrA protein leads to decreased virulence in many pathogenic bacteria (Skorko-Glonek et al., 2013). Zhu et al. (2015) constructed a mutant of *S*. Paratyphi A by deleting *htrA* and *yncD*. The double deletion mutant was completely inhibited when grown at 46°C, while the wild-type and the *yncD* mutant grew well at the same temperature. Moreover, when exposed to hydrogen peroxide, the growth of the double deletion mutant was strikingly decreased relative to the wild-type and the *yncD* mutant (Zhu et al., 2015). The virulence of the double deletion mutant was reduced by 1.58 × 10^5^ times compared with the parent strain. Nasal immunization of the attenuated strain protected immunized mice against the lethal challenge of the wild-type strain (Zhu et al., 2015).

#### *guaBA* and *clpX*

The *guaB* and *guaA* genes encode enzymes that play key roles in the *de novo* synthesis of guanine nucleotides, and therefore contribute to the regulation of bacterial growth. The *clpX* gene encodes a chaperone ATPase involving in *Salmonella* virulence (Wahid et al., 2019). Levine et al. (1999) deleted *guaBA* and *clpX* from *S*. Paratyphi A ATCC 9150 to make a mutant named CVD 1902 (Gat et al., 2011). Animal experiments showed that CVD 1902 was attenuated, immunogenic and protective against lethal challenge with the wild-type strain. Phase 1 clinical trial in volunteers demonstrated that single doses as high as 10^9^ and 10^10^ CFU were well tolerated, and elicited cell mediated immune responses against *S*. Paratyphi A (Wahid et al., 2019).

#### sptP

SptP is one of effectors of *Salmonella* Type III secretion system 1, and regulates the biogenesis of intracellular replicative niches through dephosphorylation of valosin-containing protein (Humphreys et al., 2009). A *sptP* deletion strain of *S*. Paratyphi A was assessed for its virulence, immunogenicity and protective efficacy (Pan et al., 2020). The LD_50_ of the *sptP* deletion mutant was 1.43 × 10^4^ times higher than that of the wild-type. Intraperitoneal immunization elicited cellular and humoral immune responses, and achieved a full protection against the lethal intraperitoneal challenge of the parent strain in mice.

### Subunit Vaccine Candidates

Many surface or secreted components of *Salmonella* cells, such as O-polysaccharide, outer membrane proteins and type III secretion system proteins, are excellent immunogens (Baliban et al., 2020). Several homologous components of *S*. Paratyphi A have been evaluated in mice model, demonstrating their immunogenicity and protective efficacy.

#### Outer Membrane Proteins

Yang et al. (2012) revealed that antiserum against the entire outer membrane proteins of *S*. Paratyphi A had bactericidal activity *in vitro*. The outer membrane proteins with immunogenicity were screened with immunoproteomic technology. Altogether twelve immunogenic outer membrane proteins of *S*. Paratyphi A were identified. From them, LamB (a maltoporin), PagC (a vesicle regulator), TolC (outer membrane channel), NmpC (a porin protein) and FadL (a long-chain fatty acid transport protein), which were intraperitoneally immunized at a dose of 100 μg, showed significant immunoprotection against *S*. Paratyphi A with protection rates of 95, 95, 85, 80, and 70%, respectively. Passive immunization with antisera against recombinant outer membrane proteins also conferred significant immunoprotection in mice (Yang et al., 2012).

#### SpaO and H1a

SpaO is an outer membrane protein that plays a key role in bacterial adherence and invasion (Hueck, 1998). H1a is the flagellum antigen of *S*. Paratyphi A. The encoding genes for both proteins are widely distributed in isolates of *S*. Paratyphi A (Ruan et al., 2008). Subcutaneous immunization with 100–500 μg of recombinant SpaO achieved 58.3–66.7% of protection rates in mice. Immunization with recombinant H1a achieved 41.7–58.3% of protection rates. When the mice were immunized with the mixture of SpaO and H1a, the protection rate increased to 83.3–91.7%. Furthermore, recombinant SpaO of *S*. Paratyphi A elicited cross-protection against challenges of *S*. Typhi and *S*. Schottmuelleri, respectively. However, immunization with recombinant H1a did not achieve adequate cross-protection (Ruan et al., 2008).

### Conjugate Vaccine Candidates

*S*. Typhi is enveloped by the Vi capsular polysaccharide, which serves as not only an important virulence factor but also a protective antigen (Hu et al., 2017). Vi polysaccharide vaccine is currently licensed and is used for preventing infection caused by *S*. Typhi. Due to lack of immune memory in the immune responses elicited by the Vi polysaccharide, conjugated Vi vaccines, which are produced by linking various T-dependent proteins to the Vi polysaccharide, are now assessed in clinical trials.

However, the Vi polysaccharide is deficient in *S*. Paratyphi A, whose surface is covered by the O-specific polysaccharide, a protective antigen for *Salmonella* (Konadu et al., 1996). Multiple conjugate vaccines composed of the O-specific polysaccharide of *S*. Paratyphi A bound to various carriers have been assessed for their safety and immunogenicity in animal experiments or clinical trials.

#### O-Specific Polysaccharide-Tetanus Toxoid (OSP-TT) Conjugates

The OSP-TT conjugates were developed by Konadu et al. (1996). The OSP was activated with 1-cyano-4-dimethylaminopyridinium tetrafluoroborate and linked to tetanus toxoid with or without a spacer. The resultant conjugates elicited high levels of anti-LPS IgG with bactericidal activity in mice (Konadu et al., 1996). The conjugates were then assessed in Vietnamese adults, teenagers, and 2-to 4-year-old children for their safety and immunogenicity (Konadu et al., 2000). The results showed that no significant side effects were observed in the vaccinees. At 6 weeks following injection, more than fourfold rises had been observed in the IgG anti-LPS titers in 75% of the adults, 85% of the teenagers and 90% of the children. However, reinjection did not elicit a booster response in the children (Konadu et al., 2000).

#### O-Specific Polysaccharide-Diphtheria Toxoid (OSP-DT) Conjugates

Ali et al. (2014) developed OSP-DT conjugates by linking the O-specific polysaccharide of S. Paratyphi A to DT with and without an adipic acid dihydrazide linker, named OSP-AH-DT and OSP-DT, respectively. Mice were injected subcutaneously at a 4-week interval with the conjugates at a dose of 2.5 μg of OSP. The OSP-AH-DT conjugate elicited a strikingly higher anti-OSP response relative to the response to LPS alone, while the anti-OSP response to the OSP-DT conjugate was poor and less than that elicited by LPS alone (Ali et al., 2014).

#### O-Specific Polysaccharide-CRM_197_ (OSP- CRM_197_) Conjugates

Unlike tetanus toxoid and diphtheria toxoid, the carrier protein CRM_197_, a non-toxic mutant of diphtheria toxin, does not require chemical detoxification and displays more advantages over the toxoids. The OSP-CRM_197_ conjugates of *S*. Paratyphi A were generated by Micoli et al. (2012) through binding OSP to the CRM_197_. The immunogenicity of the conjugates was evaluated in a mice model. The mice were immunized subcutaneously three times, at a 2-week interval, with 200 μL/dose of either 1 or 8 μg of OSP. The immunization elicited high levels of anti-OSP IgG with strong bactericidal activity against *S.* Paratyphi A (Micoli et al., 2012).

#### Synthetic Oligosaccharide-Bacteriophage Qβ (OSP-Qβ) Conjugate

Isolation of bacterial OSP needs large-scale fermentation of pathogenic organisms, followed by detoxification to remove endotoxin. Synthesis of oligosaccharide antigens can avoid the detoxification procedure. Dhara et al. (2020) used synthetic oligosaccharides corresponding to the O- polysaccharide repeating units of *S*. Paratyphi A to generate glycocojugate by binding to a carrier, bacteriophage Qβ. The conjugate elicited high level of anti-glycan IgG antibodies in mice. Passive immunization with the antisera protected mice from the lethal challenges with *S*. Paratyphi A (Dhara et al., 2020).

### Bivalent Enteric Fever Vaccines

Typhoid fever and paratyphoid fever A are usually simultaneously prevalent in the same area. Compared with the use of two monovalent vaccines for the control of both diseases, bivalent vaccines undoubtedly have more advantages in terms of convenience, acceptance and cost.

#### An Oral Bivalent Vaccine Candidate Based on Outer Membrane Vesicles

Bacterial cells naturally release outer membrane vesicles (OMVs), which comprise outer membrane proteins, LPS and periplasmic proteins. The OMVs are highly immunogenic and are able to elicit long-term protective immune responses (Micoli and MacLennan, 2020). The OMVs have received increasing attention as a vaccine delivery platform in recent years. Howlader et al. (2018) developed an oral bivalent typhoid vaccine by mixing the OMVs from *S*. Typhi and *S*. Paratyphi A in a 1:1 formulation. Oral immunization of BALB/c mice with the bivalent OMVs at a 2-week interval elicited strong responses of mucosal immunity, humoral immunity and cell-mediated immunity. The vaccination protected the immunized mice from the lethal challenges of *S*. Typhi as well as *S*. Paratyphi A (Howlader et al., 2018).

#### A Live Attenuated *S*. Paratyphi A Recombinationally Producing Vi Polysaccharide

Vi capsular polysaccharide is the primary protective antigen of *S*. Typhi, while it is deficient in *S*. Paratyphi A. Xiong et al. (2017) constructed a Vi-producing strain of *S*. Paratyphi A by cloning the *viaB* locus into the chromosome. The *viaB* locus was stably maintained despite more than 200 passages, and produced immunogenic Vi polysaccharide. Nasal vaccination of the engineered *S*. Paratyphi A strain significantly protected mice against the lethal challenge of *S*. Paratyphi A or *S*. Typhi.

#### *S*. Paratyphi A Outer Membrane Vesicles Displaying Vi Polysaccharide

General modules for membrane antigens (GMMA) are OMVs naturally shed by Gram-negative bacteria specifically engineered to increase blebbing and reduce endotoxin (Mancini et al., 2021). Gasperini et al. (2021) recently engineered a GMMA-producing strain of *S*. Paratyphi A that displayed the Vi polysaccharide of *S*. Typhi by introducing the pDC5-viaB plasmid. The GMMA elicited strong humoral immune responses against both Vi and O:2 with bactericidal activities (Gasperini et al., 2021). C57BL/6 mice were immunized subcutaneously with the purified vesicles at a dose of 0.5 μg. Similar levels of anti-O:2 IgG were elicited by GMMA with or without Vi, indicating the surface display of Vi did not interfere humoral immune responses against OAg. Immunization with GMMA displaying Vi also elicited anti-Vi antibodies with serum bactericidal activity, indicating its potential application in the control of enteric fever.

#### A Bivalent Vaccine Comprising of an Attenuated *S*. Typhi Strain and Its Derived Strain Expressing Flagellin H:a and Lipopolysaccharide O:2 of *S*. Paratyphi A

Soulier et al. (2021) engineered an attenuated *S*. Typhi strain expressing two predominant antigens of *S*. Paratyphi A, LPS O:2 and flagellin H:a. A bivalent vaccine was produced by combining the engineered strain and its parental *S*. Typhi strain at a ratio of 1:1 (Soulier et al., 2021). Mice were vaccinated subcutaneously with the bivalent vaccine. The immunization induced robust IgG responses against LPS O:9 as well as O:2 with promising potential for the prevention of enteric fever. The bivalent vaccine candidate is currently assessed in clinical trial (Soulier et al., 2021).

Both antibody responses and protective cellular immunity are essential for the immunoprotection against systemic *Salmonella* infections including enteric fever. Live attenuated vaccines, which are able to elicit high levels of both humoral immunity and cell-mediated immunity, are suitable for the people lacking the background of invasive-salmonella-specific cellular immunity, for example, travelers from non-epidemic area to epidemic area, and workers handling sewage, garbage, or potentially contaminated medical specimens in non-epidemic area. However, live attenuated vaccines can cause lethal infections in immunocompromised hosts, raising safety concerns for application of live attenuated vaccines in epidemic areas of systemic *Salmonella* infections, where are concurrently threatened by HIV or malaria (Gordon, 2011; Mastroeni and Rossi, 2020). Moreover, live attenuated vaccines have potential biosafety concerns, and must be evaluated with caution. For example, development of a live attenuated vaccine candidate WT05, which had been evaluated in humans, was terminated due to the prolonged fecal shedding of vaccine bacteria (Hindle et al., 2002). Therefore, non-living vaccines are more ideal choices relative to live vaccines for the population in epidemic areas. Although non-living vaccines mainly induce protective antibody responses, they confer adequate immunoprotection in the presence of background cellular immunity in the majority of the population due to low level pre-exposure to pathogenic *Salmonella* (Mastroeni and Rossi, 2020).

There are few data associated with the cross protection against paratyphoid B and C in the above studies. Given the high similarity between the typhoid *Salmonella* strains and the existence of common antigens, the cross reaction of humoral immunity, cellular immunity or mucosal immunity elicited by paratyphoid A vaccines against paratyphoid B and C can be expected. However, whether these cross reactions can lead to adequate cross protection needs to be clarified in the future.

## Conclusion and Future Prospect

Although the global incidence is gradually decreasing, the overall burden of enteric fever is still high in many parts of the world. Moreover, clinical treatment of enteric fever is challenged by the emergence and rapid spread of anti-microbial resistant strains. Enteric fever is still a global public health threaten that needs high attention and prompt action.

The control of enteric fever requires the implement of comprehensive measures, including health education, sanitation improvements, supply of clean water, advances in diagnosis and treatment, as well as programmatic use of vaccines. Vaccination is one of inexpensive and effective means.

Considering the increasing trend of paratyphoid A fever and lack of cross protection of the currently licensed vaccines, the development of paratyphoid A vaccine is imperative.

Bivalent vaccines covering the major pathogenic serovar, *S*. Typhi and *S*. Paratyphi A, are more advantageous than monovalent vaccines. Compared with monovalent vaccine of enteric fever, bivalent vaccine against both of typhoid and paratyphoid A can more effectively reduce the incidence of enteric fever, especially in high incidence areas of paratyphoid A. Moreover, the monovalent paratyphoid A vaccine is unlikely to be brought into the national immunization program, while the bivalent vaccine will be better accepted by health policy makers. Compared to separate inoculation with two monovalent vaccines, bivalent vaccines are more acceptable. The application of bivalent vaccines has more cost advantage over that of two monovalent vaccines, and therefore, is more attractive to manufacturers. Development of bivalent vaccines against enteric fever should receive greater attention in the future.

## Author Contributions

YC: conceptualization and supervision. LX, LM, LD, MD, and ML: writing – original draft preparation. All authors contributed to the article and approved the submitted version.

## Conflict of Interest

The authors declare that the research was conducted in the absence of any commercial or financial relationships that could be construed as a potential conflict of interest.

## Publisher’s Note

All claims expressed in this article are solely those of the authors and do not necessarily represent those of their affiliated organizations, or those of the publisher, the editors and the reviewers. Any product that may be evaluated in this article, or claim that may be made by its manufacturer, is not guaranteed or endorsed by the publisher.
